# Comment on Moshirfar et al. Accuracy of Six Intraocular Lens Power Calculations in Eyes with Axial Lengths Greater than 28.0 mm. *J. Clin. Med.* 2022, *11*, 5947

**DOI:** 10.3390/jcm12082911

**Published:** 2023-04-17

**Authors:** Ferdinando Cione, Margherita Di Stasi, Ciro Sannino

**Affiliations:** Department of Medicine, Surgery and Dentistry, Scuola Medica Salernitana, University of Salerno, 84122 Salerno, Italy; nandocione1993@gmail.com (F.C.); cirosanninodr@gmail.com (C.S.)

With great interest, we read the article by Moshirfar et al. [1] concerning the comparison of six IOL power formulas in eyes longer than 28.0 mm. We congratulate the authors, because achieving better results in the IOL power calculation both in naïve and in post-refractive surgery eyes is very important [2], but we would like to comment on some aspects of their study.

(1) We appreciate the utilization of different Axial Length (AL) adjustments for some formulas, such as Wang–Koch (WK) AL or Cooke-modified AL (CMAL). In fact, it is well known that AL measurement can be influenced by several factors [3,4,5] and some of them can cause it to be less reliable [4]. Therefore, AL adjustments could be useful to eliminate the systematic error in IOL power calculation [5]. We have some concerns regarding the WK AL adjustment used in this paper. The authors cited only the first WK AL adjustments published in 2011 [6] and they applied them to SRKT and Holladay 1 formulas, but these AL adjustments were updated by the same authors in 2018 [7]. The updated version of the WK adjustments should be applied when AL > 26.5 mm for the Holladay 1 formula and when AL > 27.0 mm for the SRK/T formula [7]. In addition, an AL adjustment using a nonlinear equation was proposed for the Holladay 1 formula [8]. We do not understand which WK adjustments were used: the authors specified “published by Wang et al. in 2017” but they only used the 2011 citation. The authors should have only utilized the updated WK AL adjustments, as recommended by the same creators of them [7,8].
(2) To evaluate the accuracy of the six formulas: Barrett Universal II, Emmetropia Verifying Optical, Hill–Radial Bias Function 3.0 Calculator, Holladay 1, Kane, and SRK/T, the authors utilized the Wilcoxon test. We wonder if they also performed a post hoc analysis, as correctly performed with Cochran’s test. Lacking a Bonferroni adjustment could lead to a type I error inflation [9].
(3) We would also comment on the number of evaluated eyes. Thirty-five eyes of the 25 patients were analyzed, meaning that in some patients one eye and in others both eyes were studied. It is well known that it should be advisable to apply specific statistical methods, such as the Bootstrap or generalized estimating equations (GEE), to have valid results when evaluating bilateral eyes [10,11]. Although the authors correctly affirmed that the measurements of bilateral eyes can potentially compound data, including this point in the study’s limitations [1], they did not perform such analysis; therefore, the results could not be considered reliable. In fact, ignoring the inter-eye correlation can lead to smaller P values when both eyes are in the same group [12]. In addition, even if it is understandable that is not easy to achieve a high sample size of eyes above 28.0 mm, it is always imperative to perform a preliminary sample size calculation [9]. Unfortunately, this evaluation is missing in this paper.
(4) The authors declared that they have followed the Hoffer and Savini recommendations [9] when utilizing Python Software to input IOL constants and biometrics data in online calculators of unpublished formulas. To tell the truth, Hoffer and Savini did recommend Python for a different purpose. In fact, even if Python is usually used for automated extraction from online calculators, they suggested utilizing this specific computer programming languages to optimize unpublished formulas [9], but the authors correctly did not perform a constant optimization because they analyzed a specific subgroup of long eyes. Therefore, the authors utilized Python in the right way, but for a different reason than Hoffer and Savini originally proposed.
